# Survival Outcome Prediction in Glioblastoma: Insights from MRI Radiomics

**DOI:** 10.3390/curroncol31040165

**Published:** 2024-04-14

**Authors:** Effrosyni I. Styliara, Loukas G. Astrakas, George Alexiou, Vasileios G. Xydis, Anastasia Zikou, Georgios Kafritsas, Spyridon Voulgaris, Maria I. Argyropoulou

**Affiliations:** 1 Department of Radiology, Faculty of Medicine, University of Ioannina, 45110 Ioannina, Greece; fay_styl@hotmail.com (E.I.S.); vgxydis@gmail.com (V.G.X.); anzikou@uoi.gr (A.Z.); margyrop@uoi.gr (M.I.A.); 2 Medical Physics Lab, Faculty of Medicine, University of Ioannina, 45110 Ioannina, Greece; astrakas@uoi.gr; 3 Department of Neurosurgery, Faculty of Medicine, University of Ioannina, 45110 Ioannina, Greece; georgioskafritsas@gmail.com (G.K.); svoulgar@uoi.gr (S.V.)

**Keywords:** glioblastoma, radiomics, diffusion, perfusion, survival

## Abstract

**Background:** Extracting multiregional radiomic features from multiparametric MRI for predicting pretreatment survival in isocitrate dehydrogenase (IDH) wild-type glioblastoma (GBM) patients is a promising approach. **Methods:** MRI data from 49 IDH wild-type glioblastoma patients pre-treatment were utilized. Diffusion and perfusion maps were generated, and tumor subregions segmented. Radiomic features were extracted for each tissue type and map. Feature selection on 1862 radiomic features identified 25 significant features. The Cox proportional-hazards model with LASSO regularization was used to perform survival analysis. Internal and external validation used a 38-patient training cohort and an 11-patient validation cohort. Statistical significance was set at *p* < 0.05. **Results:** Age and six radiomic features (shape and first and second order) from T1W, diffusion, and perfusion maps contributed to the final model. Findings suggest that a small necrotic subregion, inhomogeneous vascularization in the solid non-enhancing subregion, and edema-related tissue damage in the enhancing and edema subregions are linked to poor survival. The model’s C-Index was 0.66 (95% C.I. 0.54–0.80). External validation demonstrated good accuracy (AUC > 0.65) at all time points. **Conclusions:** Radiomics analysis, utilizing segmented perfusion and diffusion maps, provide predictive indicators of survival in IDH wild-type glioblastoma patients, revealing associations with microstructural and vascular heterogeneity in the tumor.

## 1. Introduction

Glioblastoma (GBM) is the most frequent and aggressive primary malignant brain tumor in adults, accounting for 14.5% of all central nervous system tumors and 48.6% of malignant central nervous system tumors [1]. According to the latest WHO classification, all GBMs are isocitrate dehydrogenase wild-type GBMs. Despite significant advances in surgery and chemoradiotherapy over the last few decades, the prognosis for patients with GBM remains poor. The median survival remains low at only 15 months, with approximately 40% survival in the first year post diagnosis and 17% in the second year [2,3].

In recent years, many potential prognostic molecular markers have been identified, particularly those derived from immunohistochemistry and genomic sequencing analyses, which may surpass traditional histologic features in predicting survival [4]. However, these methods require biopsies that are usually performed only once and may not be representative of the entire tumor mass. GBM is a heterogeneous tumor that changes rapidly and continuously [4,5,6]. Therefore, non-invasive prediction methods of survival in patients with GBM are needed.

MRI is the preferred imaging modality for GBM evaluation and by using conventional and advanced sequences, it provides information about the tumor’s location, shape, size, and regions of increased cellularity and vascularity [7,8]. Moreover, radiomics, which involves comprehensive analysis of large-scale data, can extract quantitative features from medical images to identify the tumor’s biological characteristics, such as size, shape, and internal heterogeneity. Statistical analysis techniques select the most informative features to build predictive models for patient outcomes, improving tumor diagnosis, treatment planning, and monitoring in a non-invasive and personalized approach to cancer care [9,10,11].

Recently, significant effort has been focused on finding survival predictors among quantitative MRI features. Radiomics can provide a large number of quantitative MRI features and disease characteristics that are difficult to identify by human vision alone. Radiomics analysis has been used to predict prognosis in GBMs using conventional sequences such as T2WI, FLAIR, T1WI, and T1CE [12]. Evidence suggests that better prognostication models can be created using radiomic features extracted from advanced multiparametric MRI sequences, such as diffusion and perfusion [13]. However, previous studies have either focused on a single tumor region or on a diverse population with different glioma grades [12,13,14,15,16].

The purpose of this study was to assess a uniform population of newly diagnosed patients with GBM and identify radiomic predictors of survival. Multiple subregions of the tumor’s diffusion and perfusion maps were be separately analyzed to explore the role of the functional and microstructural properties of these subregions in patient survival.

## 2. Materials and Methods

### 2.1. Subjects

A total of 155 patients diagnosed with glioblastoma and imaged with a 1.5 MRI unit between March 2005 and May 2015 were retrospectively included in this study. Among them, only 38 patients (mean age 62.2 ± 13.1 years; 19 men, 19 women) comprised the final training cohort for model building, satisfying the following inclusion criteria: (a) pathologically confirmed isocitrate dehydrogenase wild-type glioblastoma, according to the World Health Organization classification of tumors of the central nervous system; (b) diffusion and perfusion imaging performed at least one week before surgery; (c) good quality, artifact-free MRI images; (d) no previous history of brain tumors or brain surgery; (e) predominant non-hemorrhagic lesion; (f) no treatment before surgery; (g) complete clinical data; and (h) age over 18 years old. All patients underwent the same treatment protocol, which included gross total surgical resection, chemotherapy, and radiotherapy. A validation cohort, typically a smaller portion of the training cohort, was selected to be representative of the target population, while remaining independent of it. This was achieved by selecting patients scanned with a different MRI unit during a different time period. Specifically, 11 patients (mean age 59.3 ± 7.9 years; 6 men, 5 women) with the same inclusion criteria hospitalized between August 2017 and January 2022 and scanned with a 3T MRI unit were assigned to the validation cohort. The institutional review board of the University Hospital of Ioannina approved this retrospective study (3944/8 February 2024), and the requirement for informed consent was waived. The study was conducted according to the guidelines of the Declaration of Helsinki.

### 2.2. Imaging

The training MRI data were acquired before treatment using a 1.5 T MRI unit (Gyroscan Intera; Philips Medical Systems). The validation MRI data were acquired before treatment using a 3 T MRI unit. Both MRI protocols are presented in Data Supplement S1.

### 2.3. Image Processing

Perfusion maps (relative cerebral blood volume (rCBV), relative cerebral blood flow (rCBF), and mean transit time (MTT)) were calculated using the DSC-MRI toolbox [17]. The arterial input function was identified semi-automatically, and leakage correction was performed following the approach proposed by Boxerman et al. [18].

DTI analysis was performed using the FSL software package, released 6.0 (Oxford Centre for Functional MRI of the Brain, Oxford, UK). The standard preprocessing pipeline was followed: registration of the diffusion data to the b = 0 image; correction for eddy current distortions; and brain extraction. Diffusion maps (mean diffusivity (MD), fractional anisotropy (FA), axial diffusivity (AD), and radial diffusivity (RD)) were created after fitting a tensor model to the preprocessed data.

**Figure 1:**
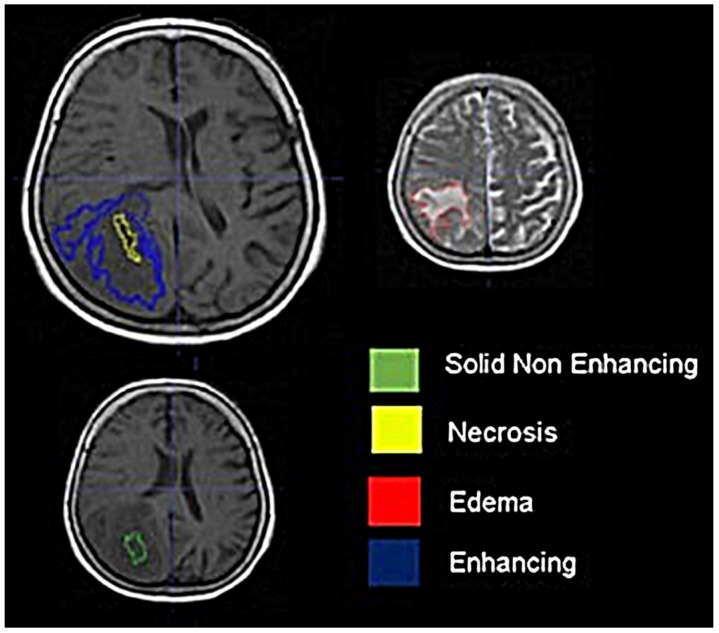
Segmentation of glioblastoma in 4 subregions using T1W (**left**) and T2W (**right**) images.

Segmentation of four tumor-related tissues (solid non-enhancing tumor tissue, enhancing tissue, necrotic tissue, and edema) was performed semi-automatically on the T1W, T1CE, T2W, and FLAIR images by a neuroradiologist (with 4 years of experience in neuro-oncological imaging) using the ITK-snap software package (Figure 1). The segmentation step produced 3D masks of each tissue co-registered with the T1W image. Then, for each subject, the T1W images were registered on the b = 0 diffusion images and on the first dynamic perfusion images using affine registration routines from the statistical parametric map software package. The calculated affine transformations were applied on the tissue masks producing new tissue masks co-registered with the diffusion and the perfusion maps which defined the regions of radiomic analysis.

### 2.4. Radiomics

For each tissue type and for each diffusion and perfusion map, radiomic features were extracted using the Pyradiomics package [19]. Due to their high resolution, the T1W images were used to extract 14 3D shape features. The 18 first-order intensity features and 68 second-order texture features (22 from gray-level co-occurrence matrix (glcm), 16 from gray-level run-length matrix (glrlm), 16 from gray-level size-zone matrix (glszm), and 14 from gray-level dependent matrix (gldm)) were extracted for three tissue types (solid, enhancing, and edema) and each calculated map (MD, FA, AD, RD, relative CBV, relative CBF, and MTT). Necrotic tissue, although visible, was not included as a separate tissue type in the second-level radiomic analysis because, very often for necrotic voxels, reliable values of the perfusion and diffusion metrics could not be calculated. For each subject, a total of 1862 different radiomic features were extracted.

### 2.5. Statistical Analysis

Statistical analyses were performed using software packages of the R statistical software (R version 4.2.1, R Core Team, Vienna, Austria). Sex was not included in the statistical analysis because there were no differences in the Kaplan–Meyer survival curves between men and women (log rank *p* = 0.295) Two methods were employed to reduce the dimensionality of the features before conducting multivariate survival analysis:

Elimination of highly correlated features: The first step involved removing highly correlated features. This is a common practice to avoid multicollinearity, which can lead to unstable estimates in regression models. A threshold of 0.7 was used to determine which features were highly correlated. This means that if the correlation coefficient between two features exceeded 0.7, one of them was removed to retain only one representative feature from each highly correlated group. The findCorrelation function from the caret package was used for this purpose. After eliminating highly correlated features, 66 uncorrelated radiomic features remained.

Univariate Cox regression analysis: In the second step, univariate Cox regression analyses were performed on the remaining uncorrelated radiomic features. Cox regression analysis is commonly used in survival analysis to assess the relationship between covariates (features) and survival time while accounting for censoring. The coxph function from the Survival package was utilized for this analysis. Each feature was assessed individually to determine its significance as a predictor of survival. Only features found to be significant predictors of survival were retained for further analysis. In this case, 25 significant features were identified through the univariate Cox regression analysis.

The final survival analysis was performed using age along with the 25 radiomic features as covariates in a multivariate Cox proportional-hazards model with LASSO least absolute shrinkage and selection operator) regularization, a method that has been reported to reliably predict survival in glioma. LASSO Cox regression analysis uses a penalty function forcing noninformative regressors to zero and leading to a more refined model [20]. To determine the optimal regularization parameter (λ) for the model, a systematic and reproducible approach was employed using 10-fold cross-validation, as implemented by the cv.glmnet function from the glmnet package in R. Recognizing the potential for variability in cross-validation outcomes, the robustness of the λ selection process was enhanced by repeating the 10-fold cross-validation procedure 100 times. This repetition aimed to mitigate the effects of randomness that can occur when partitioning the data during each cross-validation fold, thereby providing a more stable and reliable estimate for the optimal λ. The choice of λ for the final model was based on the performance metric of Harrell’s concordance measure (C-Index), a suitable and widely accepted method for assessing model performance in survival analysis. By selecting the λ that achieved the best average C-Index across all iterations of the cross-validation process, it was ensured that the model’s regularization parameter was optimized to enhance predictive accuracy while minimizing overfitting. The final model’s performance was validated internally (package: hdnom, functions: validate) by time-dependent AUC (area under the receiver operating characteristic curve) with bootstrap resampling (1000 times), every 6 months from 6 months to 30 months. Similarly, the model’s performance was validated externally with the validation dataset (package: hdnom, functions: validate_external). A nomogram was constructed as an easy-to-use prognostication model of survival. For all statistical tests a *p* value less than 0.05 was considered statistically significant.

## 3. Results

There were three survivors in the training cohort and two survivors in the validation cohort. The median follow-up times and the median survival were 12 months in the training set and 15 months in the validation set.

The final Cox proportional-hazards model had age and six radiomic features with nonzero coefficients (Table 1). Shape and first and second order radiomic features contributed to the model from T1W, diffusion, and perfusion maps. The C-Index of the model was 0.66 (95% C.I. 0.54–0.80).

The radiomic features in the final model (Table 1) reflected different aspects of the tumor microenvironment, including its shape, texture, and heterogeneity, which are known to be important factors in tumor growth and progression [10], (Data Supplement S2).

**Figure 2:**
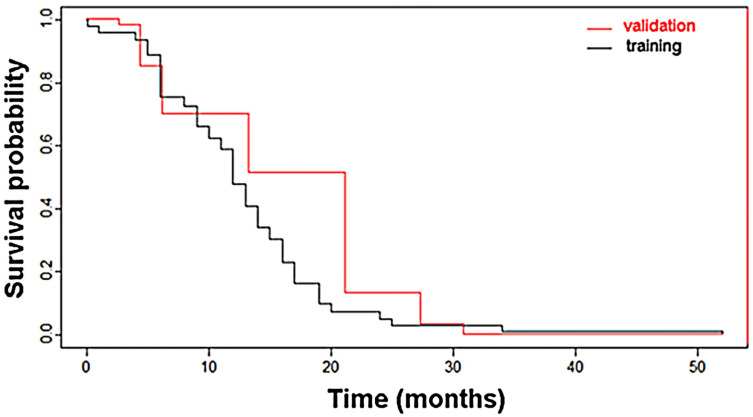
Survival curves of the validation (red line) and training (black line) datasets according to the Cox proportional-hazards model.

Survival curves produced by the model for the training and the validation datasets are shown in Figure 2.

**Figure 3:**
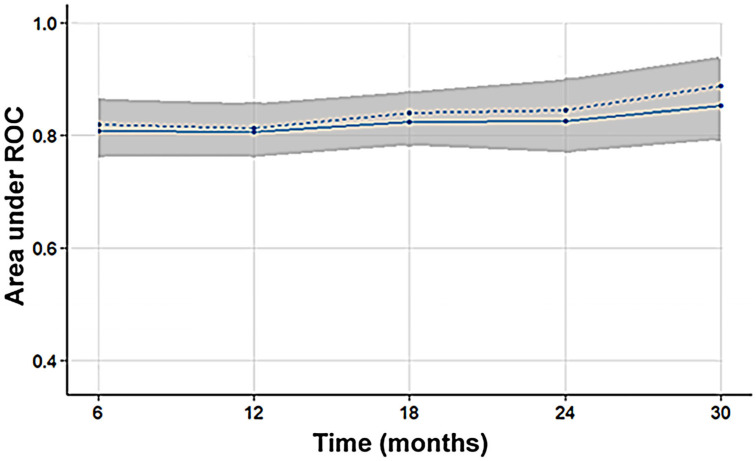
The mean (solid line), median (dashed line), and 25–75% quantiles (gray area) of the area under the receiver operating characteristic curve are shown at five time points across 1000 bootstrap predictions, indicating the internal validation of the survival model. The high area under the receiver operating characteristic curve values, around 0.8, demonstrate that the model’s predicted probabilities of survival align well with the actual survival outcomes at different time points.

**Figure 4:**
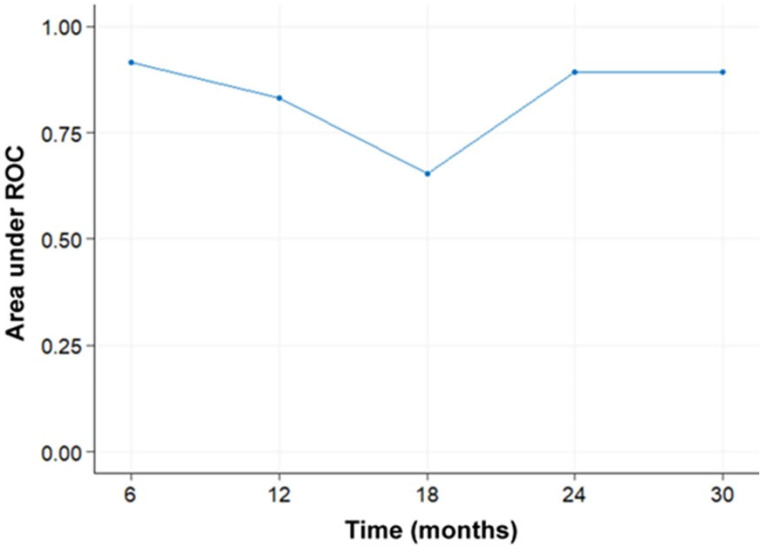
Results from the external validation of the survival model indicate accurate prediction of survival in the validation dataset, assessed by the area under the receiver operating characteristic curve at five time points. The relatively lower performance around 18 months can be attributed to the limited representation of this specific time point in the validation dataset.

The bootstrap-based internal validation results indicated a stable survival model (Figure 3). The median and the mean AUC values at each evaluation time point were close and above 0.8. The 25% and 75% quantiles were also close to the median indicating small variability. The external validation results showed that the model predicted survival with good accuracy (AUC > 0.65) at all time points (Figure 4). It performed better in the time intervals below 12 months (AUC > 0.83) and above 24 months (AUC > 0.89) and worse around 18 months. This could be explained by the small size of the validation dataset combined with poor representation of the time interval from 12 to 24 months (one case).

**Figure 5:**
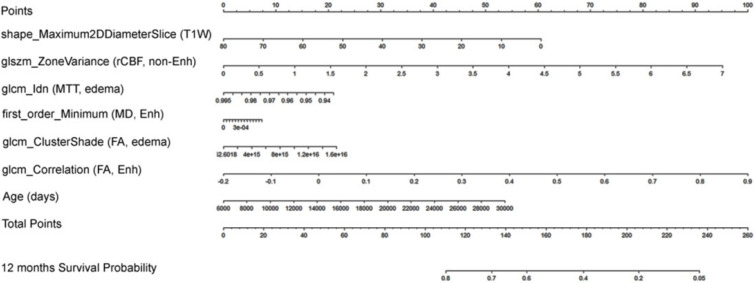
A graphical tool (nomogram) predicting the probability of 12-month survival in patients with glioblastoma. To utilize the nomogram effectively, one begins by identifying the value of each predictor on the corresponding scale. Subsequently, a vertical line is drawn from each value to the points scale (first line), determining the number of points for each predictor. After repeating this process for all predictors, the points are summed to obtain a total score. Next, this total score is located on the “Total Points” scale and a vertical line is drawn from this value to the “12 months Survival Probability” scale (last line). The point of intersection provides the estimated probability of 12-month survival for a patient based on the given predictor values.

Based on the survival model a nomogram was created as a visual tool for calculation of the one-year survival probability of GBM patients (Figure 5).

## 4. Discussion

This MRI study using radiomics analysis of perfusion and diffusion maps, demonstrated radiomic features predictive for assessing survival in patients with glioblastoma. These radiomic features are derived from various components of the tumor, including edema, necrotic regions, enhanced areas, and solid non-enhanced regions.

The population included in this study was uniform and consistent with the latest WHO classification, in contrast with previous studies that explored the prognosis of glioblastoma according to the old classification. Previous studies often included cases without the isocitrate dehydrogenase mutation, resulting in a mixture of Grade 3 and Grade 4 cases, which introduced variability in the survival assessment. This study was a notable departure from previous research that investigated the prognosis of glioblastoma based on the outdated classification system [16,21,22].

The results of the present study indicate that predictive indices of survival in patients with GBM include both perfusion and diffusion indices, highlighting the significant role of microstructural or hemodynamic characteristics in determining patient outcomes. Unlike conventional MRI protocols based on T1 and T2 imaging, which are commonly used for diagnosis and treatment, advanced MRI techniques such as diffusion and perfusion imaging have demonstrated high sensitivity in distinguishing gliomas, monitoring therapy, and predicting survival [23]. Perfusion imaging provides valuable information about the vascularization of GBM, while diffusion imaging is capable of detecting changes in cellularity and tumor cell density.

Most of the previous survival studies have primarily focused on either the whole tumor volume or the enhanced component [14,24,25,26]. However, a recent study by Zang et al. [27]. demonstrated the importance of the non-enhancing component as a high-risk subregion with implications for survival. In our study, we aimed to evaluate radiomic features extracted from all subregions of malignancy as potential predictors of survival, and interestingly, all of these features contributed to the survival modeling. This finding aligns with the study by Wu et al. [28]. which emphasized the significance of the relationship between survival and the visual characteristics of the tumor on MRI. Wu et al. demonstrated that factors such as the extent of peritumoral edema and the presence of necrosis play a crucial role in predicting the clinical outcome of glioblastoma patients. Furthermore, previous studies have consistently shown that the presence of peritumoral edema is associated with higher tumor grade and poorer survival outcomes [29,30]. Radiomic markers have been investigated within the area of peritumoral edema, and a radiomic signature of infiltration in peritumoral edema has been discovered, which can predict subsequent recurrence in glioblastoma [31,32]. Moreover, it has been established that the non-enhanced subregion of the tumor is just as cellular as the enhanced subregion [33]. Patients who undergo resection of both the non-enhanced and enhanced subregions appear to have a better prognosis [34].

In our study, shape and first and second order radiomic features contributed to the prediction model from T1W, diffusion, and perfusion maps, (Table 1). The radiomic features (shape_Maximum2DDiameterSlice, glszm_ZoneVariance, glcm_Idn, firstorder_Minimum, glcm_ClusterShade, and glcm_Correlation) in the final model reflected different aspects of the tumor, including its shape, texture, and heterogeneity, which are known to be important factors in tumor growth and progression [21].

The first feature, shape_Maximum2DDiameterSlice, was extracted from the T1CE images, specifically from the necrotic subregion. Necrotic areas within a GBM are a common imaging feature and are generally considered indicative of rapid growth and malignant behavior [28]. The negative coefficient of this feature suggests that a smaller 2D maximum diameter within a slice of the necrotic subregion is associated with a worse prognosis. This finding may initially appear contradictory when compared with previous research. Indeed prior studies, utilizing post-contrast T1-weighted MRI, have indeed recognized the necrotic subregion as a significant characteristic of glioblastoma. Hammoud et al. reported that tumors with large areas of necrosis generally exhibit poor prognoses [35]. Similarly, Lacroix et al. found that patients with small areas of necrosis who underwent aggressive resection showed significantly longer survival times [36]. However, aggressive GBMs often exhibit extensive necrosis scattered across multiple areas, resulting in a pattern associated with smaller maximum diameters within a slice [37]. Therefore, our necrotic index represented the pattern of necrosis rather than its extent, and it did not conflict with the existing literature.

The second and third features, glszm_ZoneVariance and glcm_Idn, were extracted from perfusion parametric maps specifically within the solid non-enhancing and edema subregions. These features demonstrated that inhomogeneous vascularization in the solid non-enhancing subregion and edema was associated with poor survival. The presence of inhomogeneous vascularization observed in these subregions, as indicated by glszm_ZoneVariance and glcm_Idn, served as an indicator of a poor prognosis. GBM is known to be a highly vascularized tumor; however, the blood vessels within the tumor exhibit abnormalities such as tortuosity, abnormal walls, and thrombosis. Consequently, these vessels are unable to adequately supply oxygen and nutrients to the surrounding tissue, resulting in hypoxia. Hypoxia, in turn, is associated with a poor response to treatment and increased aggressiveness, ultimately leading to decreased survival [38].

The last three features, firstorder_Minimum, glcm_ClusterShade, and glcm_Correlation, were extracted from diffusion maps, particularly from the enhancing and edema subregions. These features indicated that tissue damage in these subregions was associated with poor survival. Glioblastoma is characterized by its rapid growth and invasive nature, leading to the destruction of healthy brain tissue in the surrounding area. Diffusion techniques in MRI can detect alterations in the movement of water molecules within the brain tissue, providing valuable insights into the microstructural integrity of the tissue. Tissue damage occurring within the enhancing and edema subregions of glioblastomas can disrupt the diffusion patterns of water molecules, indicating the presence of cell death and disrupted tissue architecture. The enhancing region of a glioblastoma refers to the area where cancer cells actively divide and develop new blood vessels to support their growth. On the other hand, the edema region refers to the surrounding area where cancer cells infiltrate and disrupt normal brain tissue [39,40]. The damage inflicted on the surrounding tissue is strongly associated with poor survival, as it reflects the aggressive nature of GBM and its ability to infiltrate and destroy healthy brain tissue. Furthermore, such tissue damage can pose significant challenges in effectively treating the cancer with standard therapies such as surgery, radiation, and chemotherapy [2,41,42]. Furthermore, in our study population, we found that age was an independent prognostic factor for survival. We identified a positive correlation, indicating that as age increases, survival decreases. This is consistent with previous studies that have demonstrated that an age of 50 years or less is a significant favorable predictor of survival [43,44].

This study had several limitations that should be acknowledged. The cohort size was relatively small due to strict inclusion criteria aimed at homogenizing our groups. However, it was designed, and should be considered, as an exploratory study on the ability of radiomics to predict glioblastoma survival based on radiomics applied to multiple tumor segments using advanced MRI techniques. Another limitation was that the training dataset was not obtained from multiple sites using modern 3T MRI units or various protocols, which could have ensured comprehensive representation and generalizability of findings across different imaging setups and techniques. Nevertheless, our model’s performance was comparable to previous models, indicating the potential of our approach. Given these limitations, the results should be replicated in larger cohorts before generalization.

## 5. Conclusions

In newly diagnosed wild-type GBM, MRI radiomic features derived from various components of the lesion on diffusion and perfusion parametric maps can predict survival in a non-invasive manner. This finding emphasizes the potential clinical value of radiomics in prognostication and risk stratification of GBM patients.

## Figures and Tables

**Table 1:** Variables with non-zero coefficients contributing to the final LASSO Cox proportional-hazards model of survival prediction.

Regressor (Significant Predictor)	Tissue	Image	Regression Coefficient
shape_Maximum2DDiameterSlice	Necrosis	T1	−1.56
glszm_ZoneVariance	Non-enhancing	Relative CBF	+2.81
glcm_Idn	Edema	MTT	−7.23
firstorder_Minimum	Enhancing	MD	+2.32
glcm_ClusterShade	Edema	FA	+2.78
glcm_Correlation	Enhancing	FA	+1.87
Age			+4.62

Note: glcm, gray-level co-occurrence matrix; ldn, inverse difference moment; glszm, gray-level size-zone matrix; FA, fractional anisotropy; CBF, cerebral blood flow; MTT mean transit time; MD, mean diffusivity.

## Data Availability

Data are available on request due to restrictions. The data presented in this study are available on request from the corresponding author.

## Supplementary Materials

The following supporting information can be downloaded at: https://www.mdpi.com/article/10.3390/curroncol31040165/s1, Data Supplement S1: MRI protocols, Data Supplement S2: Radiomic features.
