# Supplementary material for: Survival Outcome Prediction in Glioblastoma: Insights from MRI Radiomics
Source: Curr Oncol. 2024 Apr 14;31(4):2233–43. doi: 10.3390/curroncol31040165 (PMC11048751; doi:10.3390/curroncol31040165)
Supplement: Supplementary file 1 [file curroncol-31-00165-s001.zip › curroncol-2932182-supplementary.pdf]

Data Supplement S1: MRI protocols

A. MRI protocol for 1.5T MRI unit (Gyroscan Intera; Philips Medical Systems):
   1. a T2W turbo spin echo, (TR/TE: 3,000/90, matrix: 250×250, field of view (FOV): 230x230 mm, slice thickness: 6 mm, gap: 0.6 mm).
   2. a FLAIR sequence (TR/TE: 6300/120, inversion recovery time: 2150 ms, FOV: 250 x250 mm, matrix: 250×250, slice thickness: 6 mm, gap: 0.6 mm).
   3. a T1W high resolution (1×1×1 mm^3) three-dimensional spoiled gradient-echo sequence (TR/TE: 25/4.6, matrix: 256×228, FOV: 220x220mm) with and without intravascular Gd injection, which was used for structural imaging.
   4. a T2* gradient echo, multi-shot EPI sequence (TR/TE: 702/30, FOV: 250x250 mm, matrix: 128×128, slice thickness: 7 mm, gap: 0, dynamic scans: 50, imaging time per dynamic scan: 2.1 s), 0.1 mmol/kg gadolinium is typically injected via an 18-gauge intravascular catheter at 5 cc/s, which was used for perfusion imaging.
   5. a single-shot spin-echo echo-planar sequence (TR/TE: 9807/131 ms, FOV: 230 x230 mm, matrix: 128×128, section thickness: 3 mm, maximum b-value: 1000 s/mm^2, 16 noncollinear diffusion directions, intersection gap: 0 mm) which was used for diffusion tensor imaging (DTI).

B. MRI protocol for 3T MRI unit (Ingenia CX; Philips Medical Systems):
   1. a T2W turbo spin echo, (TR/TE: 2.945/80, matrix: 576 × 576, field of view (FOV): 230 x 230mm, slice thickness: 4 mm, gap: 1 mm).
   2. a FLAIR sequence (TR/TE: 4800/261, inversion recovery time: 1650 ms, FOV: 251 x 251 mm, matrix: 248 × 248, slice thickness: 1 mm, gap: -0.6 mm).
   3. a T1W high resolution (1×1×1 mm3) three-dimensional spoiled gradient-echo sequence (TR/TE: 7.9/3.5, matrix: 560 × 560, FOV: 240 x 240mm) with and without intravascular Gd injection, which was used for structural imaging.
   4. a T2* gradient echo, multi-shot EPI sequence (TR/TE: 1970/40, FOV: 224 x 224mm, matrix: 128 × 128, slice thickness: 4 mm, gap: 0, dynamic scans: 50, imaging time per dynamic scan: 2s), 0.1 mmol/kg gadolinium is typically injected via an 18-gauge intravascular catheter at 5 cc/s, which was used for perfusion imaging.
   5. a single-shot spin-echo echo-planar sequence (TR/TE: 2812/80 ms, FOV: 204/204 mm, matrix: 128×128, section thickness: 2.5 mm, maximum b-value: 1000 s/mm^2, 16 noncollinear diffusion directions, intersection gap: 0 mm) which was used for diffusion tensor imaging (DTI).

Data Supplement S2: Radiomic features

Here is a breakdown of the specific radiomic features in the final model (Table 1) and their likely interpretations:

1. **shape_Maximum2DDiameterSlice**: This feature measures the maximum diameter of the tumor observed in a two-dimensional slice of the image. It provides information about the overall size and extent of the tumor within a particular plane.
2. **glszm_ZoneVariance**: It quantifies the variability in the gray-level zones present within the tumor. This feature reflects the heterogeneity within the tumor, indicating how diverse the gray-level patterns are throughout the tumor region. A higher value suggests greater heterogeneity.
3. **glcm_Idn**: This feature assesses the homogeneity of the gray-level co-occurrence matrix within the tumor. The gray-level co-occurrence matrix captures the spatial relationships between pixels of different intensities. A higher value of glcm_Idn indicates a more homogeneous distribution of gray levels within the tumor.
4. **firstorder_Minimum**: It measures the minimum intensity value observed within the tumor. This feature can provide insights into the degree of tissue damage within the tumor. A lower value indicates a potentially more damaged or necrotic region.
5. **glcm_ClusterShade**: This feature characterizes the skewness of the gray-level co-occurrence matrix within the tumor. It captures the asymmetry of the distribution of co-occurring gray-level values. Higher values suggest increased heterogeneity and spatial organization within the tumor.
6. **glcm_Correlation**: It quantifies the correlation between the gray-level co-occurrence matrix elements within the tumor. This feature reflects the degree of spatial organization and texture within the tumor. A higher value indicates stronger spatial relationships between neighboring pixels.
